# *MRP1* gene expression level regulates the death and differentiation response of neuroblastoma cells

**DOI:** 10.1054/bjoc.2001.2144

**Published:** 2001-11

**Authors:** A E Peaston, M Gardaneh, A V Franco, J E Hocker, K M Murphy, M L Farnsworth, D R Catchpoole, M Haber, M D Norris, R B Lock, G M Marshall

**Affiliations:** Children's Cancer Institute Australia for Medical Research, PO Box 81, Randwick, 2031; Centre for Children's Cancer and Blood Disorders, Sydney Children's Hospital, Randwick, NSW, 2031, Australia

**Keywords:** MRP1, neuroblastoma, apoptosis, neuritic differentiation, Bcl-2

## Abstract

We have previously reported a strong correlation between poor prognosis in childhood neuroblastoma (NB) patients and high-level expression of the transmembrane efflux pump, Multidrug Resistance-associated Protein (MRP1), in NB tumour tissue. In this study, we inhibited the endogenous expression of *MRP1* in 2 different NB tumour cell lines by stably transfecting an *MRP1* antisense expression vector (*MRP*-AS). Compared with control cells, *MRP*-AS transfectant cells demonstrated a higher proportion of dead and morphologically apoptotic cells, spontaneous neuritogenesis, and, increased synaptophysin and neurofilament expression. Bcl-2 protein expression was markedly reduced in *MRP*-AS cells compared to controls. Conversely, we found that the same NB tumour cell line overexpressing the full-length *MRP1* cDNA in sense orientation (*MRP*-S) demonstrated resistance to the neuritogenic effect of the differentiating agent, all-*trans*-retinoic acid. Taken together, the results suggest that the level of *MRP1* expression in NB tumour cells may influence the capacity of NB cells for spontaneous regression in vivo through cell differentiation and death. © 2001 Cancer Research Campaign  http://www.bjcancer.com


British Journal of Cancer (2001) 85(10), 1564–1571
© 2001 Cancer Research Campaign
doi: 10.1054/ bjoc.2001.2144, available online at http://www.idealibrary.com on IDEAL

http://www.bjcancer.com

### *MRP1* gene expression level regulates the death and differentiation response of neuroblastoma cells

**AE Peaston[1]*, M Gardaneh[1]*, AV Franco[1], JE Hocker[1], KM Murphy[1], ML Farnsworth[1], DR Catchpoole[1], M Haber[1], MD Norris[1], RB Lock[1] and GM Marshall[1,2]**

[1]Children's Cancer Institute Australia for Medical Research, PO Box 81, Randwick, 2031 and the [2]Centre for Children's Cancer and Blood Disorders, Sydney Children's Hospital, Randwick, NSW 2031, Australia

**Summary** We have previously reported a strong correlation between poor prognosis in childhood neuroblastoma (NB) patients and high-level expression of the transmembrane efflux pump, Multidrug Resistance-associated Protein (MRP1), in NB tumour tissue. In this study, we inhibited the endogenous expression of *MRP1* in 2 different NB tumour cell lines by stably transfecting an *MRP1* antisense expression vector (*MRP*-AS). Compared with control cells, *MRP*-AS transfectant cells demonstrated a higher proportion of dead and morphologically apoptotic cells, spontaneous neuritogenesis, and, increased synaptophysin and neurofilament expression. Bcl-2 protein expression was markedly reduced in *MRP*-AS cells compared to controls. Conversely, we found that the same NB tumour cell line overexpressing the full-length *MRP1* cDNA in sense orientation (*MRP*-S) demonstrated resistance to the neuritogenic effect of the differentiating agent, all-*trans*-retinoic acid. Taken together, the results suggest that the level of *MRP1* expression in NB tumour cells may influence the capacity of NB cells for spontaneous regression in vivo through cell differentiation and death. © 2001 Cancer Research Campaign http://www.bjcancer.com

**Keywords**: MRP1; neuroblastoma; apoptosis; neuritic differentiation; Bcl-2

Neuroblastoma (NB) is the most common solid tumour in early childhood and is thought to arise from primitive neural crest cells, which eventually give rise to mature sympatho-adrenal tissues. Infant necropsy studies and mass infant screening programs for NB have demonstrated a subclinical incidence of the disease, which is more than twice the clinical incidence, suggesting a high rate of spontaneous tumour regression (Beckwith and Perrin, 1963; Woods et al, 1996). Several lines of evidence indicate that a process of ganglionic differentiation may be a necessary precursor to tumour regression and neuroblast cell death (Evans et al, 1976; Shimada et al, 1984). Taken together these observations have suggested the existence of tumorigenic factors for NB, which act by blocking normal embryonal neural crest differentiation and cell death.

We, and others, have previously shown that poor patient prognosis correlates with high-level expression of the Multidrug Resistance-associated Protein gene (*MRP1*) in primary NB tumour tissue (Norris et al, 1996; Matsunaga et al, 1998; Bader et al, 1999). MRP1 is a 190 kDa protein belonging to the superfamily of ATP-binding cassette (ABC) transmembrane transporters, such as P-glycoprotein (Pgp). Overexpression of MRP1 in vitro has been associated with low-level resistance to a wide range of cytotoxic agents, overlapping with the range of drugs to which Pgp confers resistance (Cole et al, 1992, 1994). *MRP1* knockout mice show increased sensitivity to etoposide, decreased inflammatory responses, and increased tissue glutathione (Lorico et al, 1997; Wijnholds et al, 1997). Collectively, these data suggest that, like Pgp, MRP1 functions as an export pump of cytotoxic xenobiotics (Deeley and Cole, 1997). High-level *MRP1* expression in NB tumour cells may provide protection against cytotoxic chemotherapy, thereby mediating drug resistance in vivo. However, our own studies indicated that tumour relapse was predicted by high-level *MRP1* expression in some patients with localised disease who had not received chemotherapy. This observation led us to examine the hypothesis that high-level *MRP1* expression in malignant neuroblasts may function as a resistance factor for neuritic differentiation and cell death.

### MATERIALS AND METHODS

### Cell culture

The NB cell lines BE(2)-C (BE), with amplified *MYCN*, and SHSY-5Y (SY), with single copy *MYCN*, were the kind gift of Dr J Biedler, Memorial Sloan-Kettering Cancer Center, New York, NY, USA. The human mammary carcinoma cell line MCF7WT, and its *MRP1*-amplified subline MCF7VP, were the kind gift of Dr E Schneider, Wadsworth Center for Laboratories and Research, Albany, New York, NY, USA. Cells were maintained at 37˚C, 95% humidity, 5% $CO_2$ in air, in Dulbecco's Modified Eagle's Medium (Life Technologies, Grand Island, NY, USA) supplemented with L-glutamine and 10% fetal calf serum (Trace Biosciences Pty Ltd, Sydney, Australia). Transfected cells were selected and subsequently grown with the additional supplement of 0.5 mg $ml^{-1}$ Hygromycin B (Calbiochem-Novabiochem Pty Ltd, San Diego, CA, USA).

Growth rates and evidence of morphologic differentiation of individual control and transfectant NB clones were determined

*Received 21 December 2000*
*Revised 17 July 2001*
*Accepted 19 July 2001*

*Correspondence to:* GM Marshall

*These authors contributed equally to this work.

using Trypan blue exclusion and morphologic evidence of neurite extension, as previously described (Marshall et al, 1995). Cells were treated with all-*trans*-retinoic acid (aRA) (Sigma Chemical Company, St Louis, MO, USA), 9-*cis*-retinoic acid and 13-*cis*-retinoic acid (kind gift of Hoffman LaRoche, Basel, Switzerland) to final concentrations of 0.01, 0.1, 1 or 10 μM. The percentage of apoptotic cells was estimated by examination of cell morphology as previously described (Lock and Stribinskiene, 1996).

To examine cellular cytotoxicity, BE cells overexpressing MRP1 were seeded in 96-well plates and growth inhibition determined after 72 hours continuous exposure to various concentrations of sodium arsenate (Baker, Biolab Scientific, Sydney, Australia), using a microtitre-based assay with the Alamar Blue™ reagent (Astral Scientific, Sydney, Australia). Using a log scale for molar concentration, data points from at least 2 replicate assays were fitted to a spline curve, and $ID_{50}$ values determined from the curve as previously described (Haber et al, 1989).

### Plasmid constructs and transfections

The pCEBV7-*MRP1* (CEP *MRP1*) episomal expression vector containing the full length human *MRP1* cDNA in sense orientation was the kind gift of Dr SPC Cole, Cancer Research Laboratories, Queen's University, Kingston, Ontario, Canada. CEP was derived from pREP7 (Invitrogen, San Diego, CA, USA) by substituting a cytomegalovirus promoter for the Rous sarcoma virus long terminal repeat (Cole et al, 1994). To create *MRP1* antisense constructs, CEP *MRP1* cDNA was digested with *HindIII*, to generate a 1.7 kb fragment including a short segment of the CEP polylinker and 0–1633 bp of the 5′ end of the *MRP1* cDNA, and a 1.3 kb fragment of the *MRP1* cDNA from 1634 bp to 2962 bp. After purification, each fragment was cloned into the *HindIII* restriction enzyme site in the mammalian expression plasmid pMEP, where cloned fragments are under the control of the human metallothionein $II_A$ promoter. While the human metallothionein $II_A$ promoter is inducible by heavy metals, we have found that in NB cells a low level of constitutive expression of cloned sequences also occurs. As an empty vector control for comparison with SY *MRP*-AS clones, SY cells were stably transfected with the pREP plasmid. The pREP plasmid is identical to the pMEP plasmid, except that cloned cDNAs are under the control of the Rous sarcoma virus long terminal repeat, instead of the human metallothionein $II_A$ promoter. In prior experiments, we have shown no difference in the growth behaviour of untransfected BE or SY cells, or BE and SY cells transfected with either the pREP or pMEP empty vector. BE and SY cells were transfected by electroporation with empty vectors (CEP, REP, MEP), or plasmids containing *MRP1* cDNA in sense (*MRP*-S), or antisense (*MRP*-AS) orientation, and transfectants were selected in Hygromycin B and perpetuated as previously described (Marshall et al, 1995).

### RNA isolation and PCR

Total cellular RNA was isolated (Chomczynski and Sacchi, 1987) and used to make cDNA using Moloney murine leukaemia virus reverse transcriptase (Life Technologies) and random hexanucleotide primers. To evaluate the relative mRNA expression levels of *MRP1* in MRP-S transfected cell lines, cDNA equivalent to 50 ng of mRNA from each line was subjected to co-amplification of target (*MRP1*) and control (*β2-microglobulin*) gene sequences. The gene-specific oligonucleotide primers for *MRP1* and the *β2-microglobulin* gene, the PCR conditions and methods of estimating gene expression by reverse-transcriptase PCR have been previously reported (Bordow et al, 1994; Haber et al, 1994). To evaluate relative expression of *Bcl-2* in MRP-AS cells, *β2-microglobulin* was the control gene. Gene-specific PCR primers for *Bcl-2* were B11S (5′ACAA-CATCGCCCTGTGGATGAC3′), and B12AS (5′AGCCAGGAG AAATCAAACAGAGG3′), which amplified a 132 bp sequence from 1970 bp to 2102 bp of the Bcl-2 coding sequence.

### Protein isolation and immunoblotting

To measure cellular MRP protein, crude membranes were prepared as previously described (Grant et al, 1994). For other immunoblotting experiments, whole cell lysates were prepared from harvested cell pellets by incubation in RIPA buffer (50 mM Tris.Cl (pH 7.5), 150 mM NaCl, 1% Nonidet P-40, 0.5% Na deoxycholate, 0.1% SDS) for 30 min on ice, with vortexing every 10 minutes. The lysate was clarified by centrifugation (14 ***g***, 10 min, 4˚C) and the supernatant stored at –70˚C until further use. Protein content was determined using the BCA Protein Assay Kit (Pierce Chemical Co, Rockford, IL, USA) and samples adjusted to equal protein content before use.

Equal sample volumes were separated by SDS-PAGE, transferred and immunoblotted using monoclonal anti-MRP1 antibody, MRPr1 (Signet Laboratories, Inc, Dedham, MA, USA), or polyclonal anti-Bax, anti-Bcl-2, anti-Bcl-$x_L$, and anti-actin antisera (PharMingen, San Diego, CA, USA). After incubation of membranes in horseradish peroxidase-conjugated secondary antibodies, immunostained bands were detected using chemiluminescent methods (ECL, Amersham International Plc, Little Chalfont, Bucks, UK; or SuperSignal Substrate, Western Blotting, Pierce Chemical Co) with image collection on X-ray film (ECL) or by phosphorimager (SuperSignal). Quantitation of immunostained bands was by densitometry (X-ray film), or by image analysis of phosphorimage data using Multi Analyst 1.02 software (Bio-Rad). Equal protein loadings were checked by immunostaining with an anti-β-actin rabbit polyclonal antibody, and, by visual inspection or densitometry of duplicate gels stained with Coomassie Blue.

### Immunocytochemistry

Cells grown on collagen-coated cover slips were fixed in 4% paraformaldehyde in PBS, pH 7.4, rinsed 3 times in PBS, then further treated with ice cold methanol for 15 minutes, followed by 2 rinses with PBS. After blocking, the cells were immunostained with monoclonal antibodies directed against either synaptophysin (anti-SVP-38 antibody, Sigma Chemical Company), or high molecular weight neurofilament (anti-NF-200 antibody, Sigma Chemical Company). Stained coverslips were mounted on slides using the ProLong Antifade Kit (Molecular Probes, Inc). The slides were examined using an LSM GB 200 laser scanning confocal microscope (Olympus, Tokyo, Japan) with excitation wavelengths of 488 nm (Argon) and 543 nm (Helium). The images were collected electronically (1024 × 768 pixels) and analysed using NIH Image software (W. Richards, National Institutes of Health, Bethesda, MD, USA). To estimate relative cellular protein content, mean pixel density of 5 images each containing 20 individual cells was calculated by measuring the mean pixel density of 20 identical sized spots per image (excluding measurements made over the nucleus) and subtracting it from the mean non-cellular background measured in each image by the same method.

© 2001 Cancer Research Campaign 

### Caspase activity assays

Cytosolic lysates were used to estimate caspase activity by cleaving the synthetic caspase-3 substrate, N-acetyl-Asp-Glu-Val-Asp-*p*-nitroanilide (Ac-DEVD-pNA) (BIOMOL Research Laboratories). Briefly, $1 \times 10^7$ cells were washed in ice cold PBS, resuspended in 1 ml lysis buffer 3 (10 mM HEPES, pH 7.4; 10 mM NaCl; 25 μg ml$^{-1}$ phenylmethylsulfonyl fluoride; 1 μg ml$^{-1}$ leupeptin; 1 μg ml$^{-1}$ aprotinin) and incubated on ice for 30 minutes. The cells were then disrupted by repeated freeze/thawing, ultracentrifuged at 100 000 ***g*** for 1 hour at 4˚C, and the resultant cytosolic (S100) and crude nuclear/membrane/organelle (P100) fractions stored separately at –70˚C after resuspension of the P100 in 1 ml lysis buffer 3. Protein content was determined using the BCA Protein Assay Kit (Pierce Chemical Co, Rockford, IL, USA), and samples adjusted to equal protein content. An aliquot of lysate equivalent to 100 μg of protein was added to 150 μl of reaction buffer (20 mM PIPES pH 7.2, 100 mM NaCl, 1 mM EDTA, 10% sucrose, 0.1% 3-([3-cholamidopropyl]dimethylyammonio)-1-propanesulfonic acid (CHAPS), 20 mM 2-mercaptoethanol) containing the peptide substrate (final concentration 100 μM) in a 96-well plate. The absorbance of each well at 405 nm (A405) was read both before and following a 1 hour incubation at 37˚C. Ac-DEVD-pNA cleavage activity was measured as A405 h$^{-1}$ mg protein$^{-1}$, and expressed as % Ac-DEVD-pNA cleavage or caspase 3 activity. Preliminary experiments had verified linearity of response over time and protein concentration (data not shown).

### TUNEL labelling

NB cells were stained using the In situ cell death detection kit (Roche, Castle Hill, Australia). Briefly, cells were harvested, along with their respective supernatants, and washed once with cold PBS. A total of $5 \times 10^8$ cells were placed on a polylysine glass slide for cytospin preparation. Following cytospin, the slides were fixed in a 4% paraformaldehyde solution for 1 hour at room temperature. The slides were subsequently rinsed with PBS and incubated in a permeabilising solution (0.1% Triton X-100, 0.1% sodium citrate) for 2 minutes at 4˚C. The slides were then rinsed twice with PBS and dried. 50 μl of the TUNEL reaction mixture was added onto the slides, which were incubated at 37˚C for one hour. The slides were subsequently rinsed 3 times in PBS before analysis under a fluorescence microscope. 5 microscopic fields (> 100 cells), in duplicate experiments, were counted in duplicate slides to determine the proportion of TUNEL positive or apoptotic cells.

### RESULTS

### Down-regulation of endogenous MRP1 expression in NB cells

The NB tumour cell lines, BE and SY, were transfected with vectors constitutively expressing, in antisense, either a 1.3 kb (1634–2962 bp) or a 1.7 kb (0–1633 bp) fragment of the human *MRP1* cDNA. Following 2 separate transfection experiments for each cell line, individual clones (*MRP*-AS) were isolated in Hygromycin B and characterised for *MRP1* expression (Figure 1A and 1B). There was a significant difference between the growth rate of BE *MRP*-AS clones, and control MEP cells (Figure 2). Compared with control cells, BE *MRP*-AS clones were markedly growth inhibited, with day 7 cell counts ranging from 15% to 39% of controls. Similarly, SY *MRP*-AS clones were markedly growth inhibited when compared with controls. Morphologic analysis of BE *MRP*-AS clones demonstrated marked spontaneous neurite formation (Figure 3). There was a strong inverse correlation between *MRP1* expression level and the proportion of BE *MRP*-AS cells with neurites after 7 days in culture when individual clones were compared (Figures 1 and 3).

Increased expression of synaptophysin and high-molecular-weight neurofilament are features of neuronally differentiated cells (Jahn et al, 1985). Confocal microscopy and image analysis

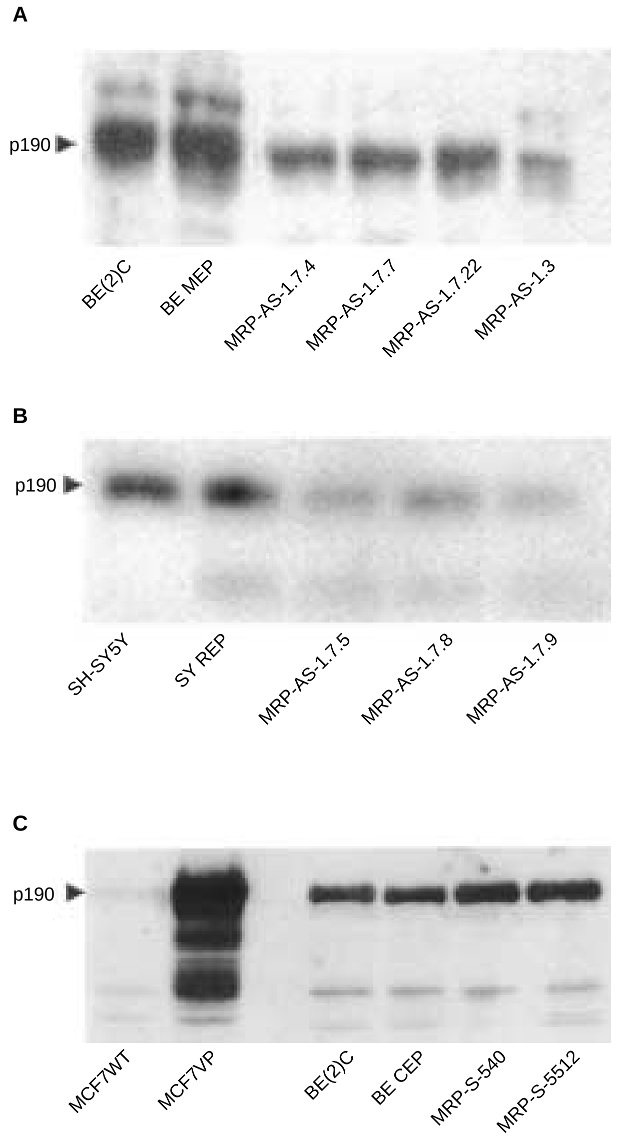

**Figure 1** MRP1 protein expression in control, *MRP*-AS (**A** and **B**) and *MRP*-S (**C**) clones for the BE(2)-C (BE) (**A** and **C**) and SH-SY5Y (SY) (**B**) neuroblastoma cell lines. In each panel control lysates from untransfected BE or SY cells, and, BE or SY cells stably transfected with empty vector (CEP, REP or MEP) have been included on the immunoblot. MRP1 protein expression in control cells has been compared with BE or SY clones stably expressing either a partial MRP1 cDNA cloned in antisense (panel **A** and **B**) or the full-length MRP1 cDNA in sense (panel **C**, clones MRP540 and 5512). Cell membrane protein lysates from parental, empty vector control, transfectant clones for BE or SY cells were size-fractionated, and then immunoblotted for MRP1 expression using an antibody specifically recognising MRP1 as described in Materials and Methods. As a control for high-level MRP1 expression in *MRP*-S transfectants, membrane lysates from MCF7 breast cancer cells (MCF7WT), and, MCF7 cells containing an amplified and overexpressed *MRP1* gene (MCF7VP) were included in panel **C**

 *© 2001 Cancer Research Campaign*

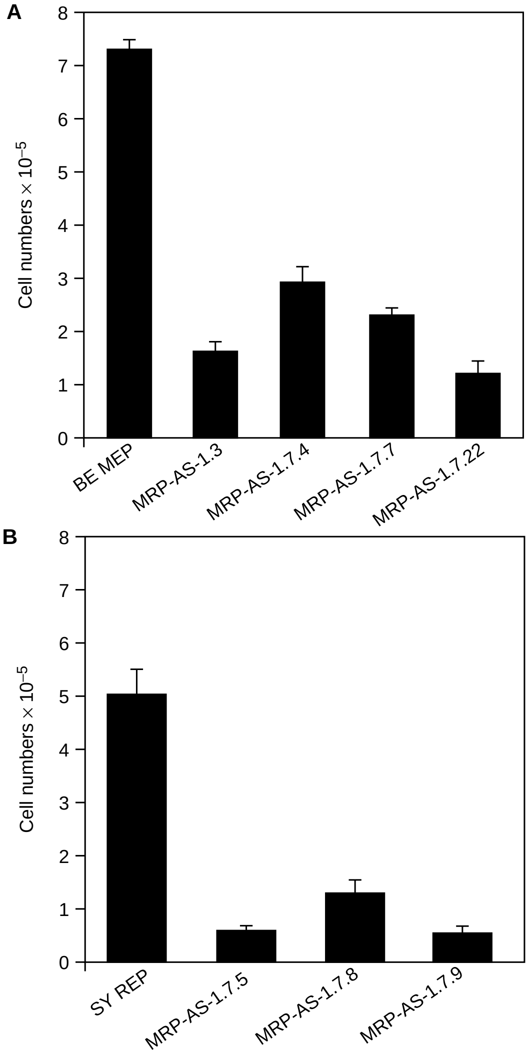

**Figure 2** Growth characteristics of *MRP*-AS and control cells. Cells were plated at equal density on day –1, cultured under standard conditions, and live cells counted at days 7 for BE (**A**) or SY (**B**) transfectant cells. Trypan blue dye exclusion was used to distinguish live cells. The values represent the mean of experiments performed three times in duplicate. Bars, standard error of mean

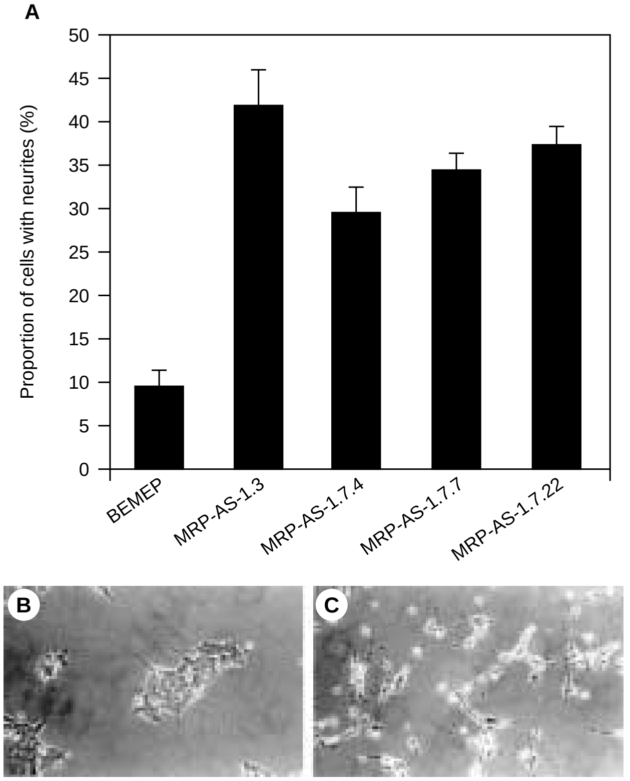

**Figure 3** Assessment of neuritic morphology of BE *MRP*-AS and control cells. Control (BE MEP) and BE *MRP*-AS cells were cultured under standard conditions for 1 week, then the proportion of cells with neurite development assessed as described in Materials and Methods. The values represent the mean of 3 experiments performed in duplicate. Bars, standard error of mean. (**A**) Histogram demonstrating the proportion of cells with neurites for control (BE MEP) and the indicated BE *MRP*-AS clones. (**B**) and (**C**), representative photomicrographs (100 × magnification) of BE MEP and *MRP*-AS clone 1.7.4, demonstrating neuritic morphology typical of *MRP*-AS clones of immunostained cells demonstrated increased expression of synaptophysin and high molecular weight neurofilament in BE *MRP*-AS cells compared with untreated control cells (Figure 4). The expression levels of synaptophysin and neurofilament in

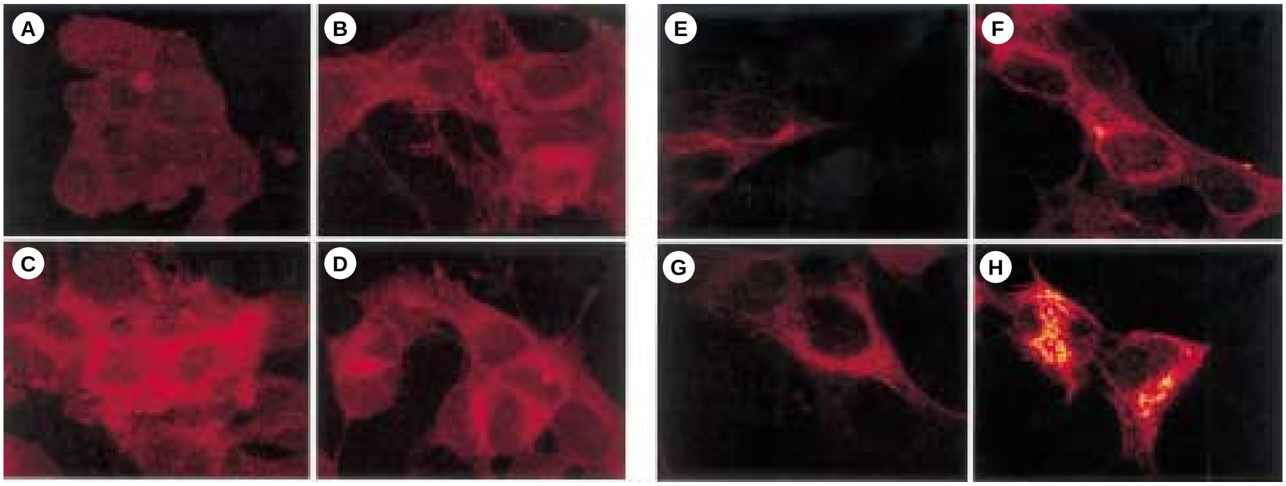

**Figure 4** Expression of synaptophysin and high molecular weight neurofilament in untreated BE *MRP*-AS clones. As a positive control for expression of synaptophysin (**A**–**D**) and neurofilament (**E**–**H**), control BE MEP cells (**A** and **E**) were cultured in the presence (**B** and **F**) of 1 μM aRA. Untreated *MRP*-AS clones 1.3 (**C** and **G**) and 1.7.4 (**D** and **H**) were immunostained for synaptophysin (**C** and **D**) or neurofilament (**G** and **H**) and examined by confocal microscopy

© 2001 Cancer Research Campaign untreated BE *MRP*-AS cells were equivalent to that seen in control cells undergoing morphologic differentiation following retinoid treatment (Figure 4).

### MRP-AS clones demonstrate enhanced spontaneous cell death

We used trypan blue staining to determine the proportion of dead cells in culture one week after plating. BE and SY *MRP*-AS clones all had a significantly higher proportion (0.5–2.5 fold) of dead cells than control cells (data not shown). Cells grown on slides in 2-well chambers were stained with Wright's stain and examined for apoptotic morphology. A proportion of *MRP*-AS cells exhibited typical apoptotic morphology (Figure 5A), and, in comparison with control MEP cells, *MRP*-AS clones had a higher proportion of morphologically apoptotic cells (Figure 5B). To confirm the presence of apoptosis in *MRP*-AS cells we evaluated TUNEL positivity and Caspase 3 activation (Figure 6). *MRP*-AS clones 1.3 and 22 exhibited a higher proportion of TUNEL-positive cells compared to controls during log phase growth.

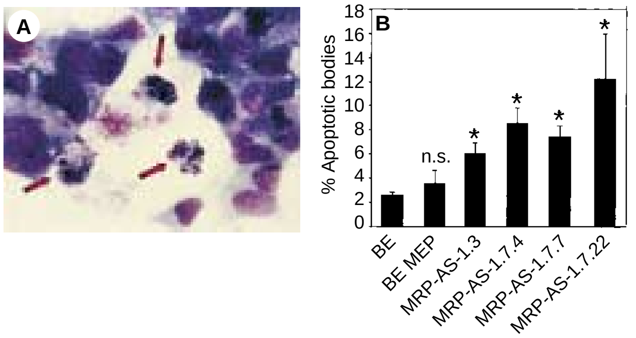

**Figure 5** Apoptosis induced by *MRP*-AS transfection. Cells grown on chamber slides were stained with Wright's stain and examined for apoptotic morphology. (**A**) Representative photomicrograph showing apoptotic BE *MRP*-AS clone 1.7.22 cells (arrows) surrounded by live cells (400 × magnification). (**B**) Histogram showing apoptotic index of parental (BE), control (BE MEP) and indicated *MRP*-AS clones. Values represent the mean of at least 6 independent determinations for each clone as described in Material and Methods. Bars, standard error of mean. Differences in the apoptotic index between control and *MRP*-AS lines were assessed by two-tailed unpaired means comparison. *$P < 0.05$; n.s., no significant difference

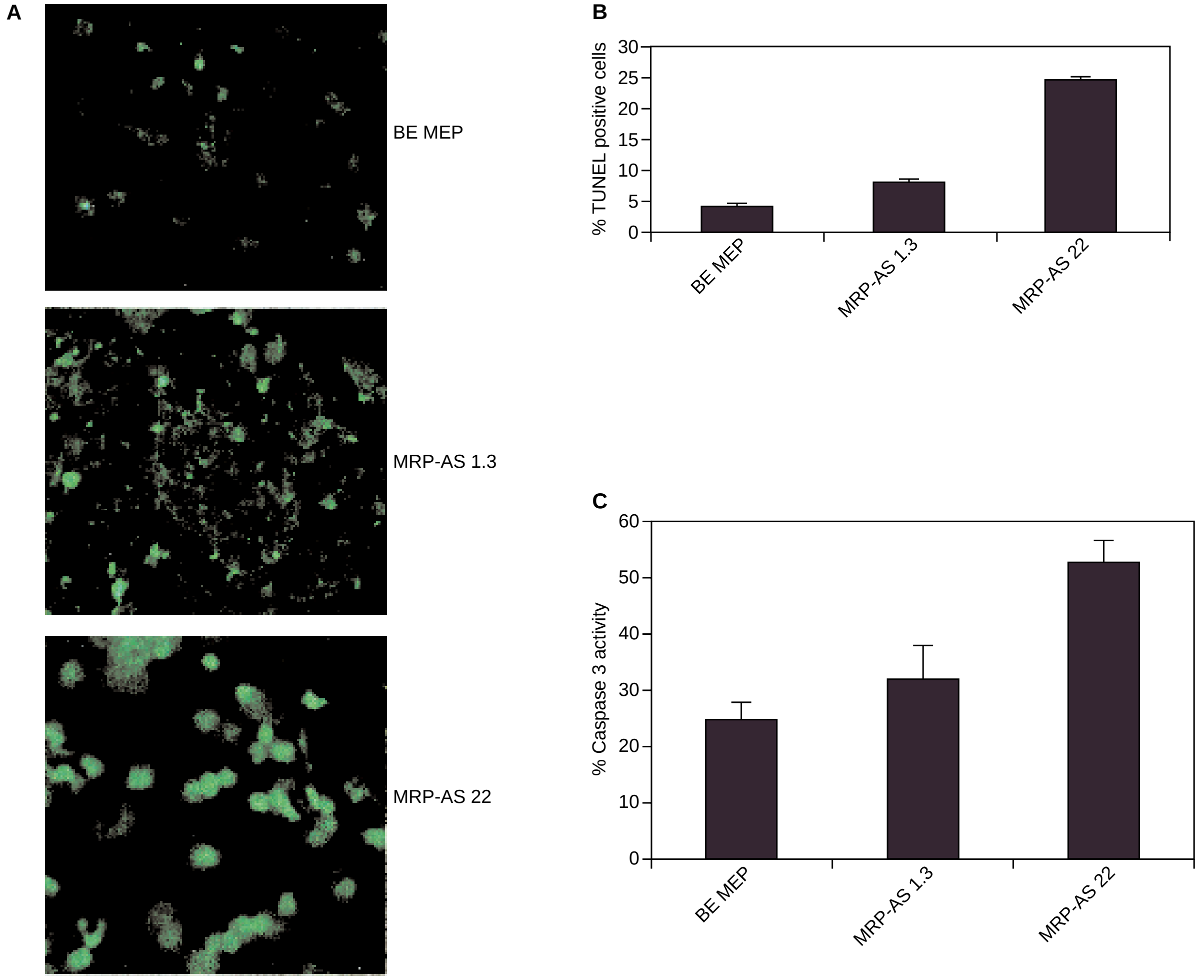

**Figure 6** TUNEL labelling and Caspase 3 activation in *MRP*-AS cells. Control (BE MEP) and BE *MRP*-AS cells were cultured under standard conditions for 1 week, then placed on a glass slide by cytospin preparation. (**A**) Representative photomicrographs showing an increase in TUNEL-positive cells among *MRP*-AS clones 1.3 and 22. (**B**) A histogram showing the proportion of TUNEL-positive cells in the same cytospin preparations. More than 100 cells were counted in 2 replicate experiments. (**C**) A histogram expressing Ac-DEVD-pNA cleavage or caspase 3 activity for *MRP*-AS clones 1.3 and 22 compared with control

© 2001 Cancer Research Campaign

The increase in TUNEL positivity was proportionately similar to the increase in apoptotic bodies shown for these clones in Figure 5B. Caspase 3 activity was similarly increased in both clones. Thus, in the *MRP*-AS transfectants, growth data, morphologic and immunocytochemical evidence showed that down-regulated MRP1 expression was associated with growth inhibition, neuritic differentiation, and apoptosis.

### Bcl-2 protein expression is decreased in MRP-AS cells

Differentiation and apoptosis have been linked to the level of Bcl-2 expression in NB primary tumour tissue and cell lines (Hoehner et al, 1995). We therefore examined Bcl-2 mRNA and protein expression levels in the *MRP*-AS and control cells. Bcl-2 mRNA expression levels were similar for control and *MRP*-AS clones (Figure 7A). However, immunoblots showed that the *MRP*-AS clones had markedly reduced Bcl-2 protein expression compared with control MEP and parental BE cells (Figure 7B). Antisera directed against Bcl-$x_L$ and Bax demonstrated no significant difference in the expression levels of these proteins for *MRP*-AS and control cells (Figure 7B). These results suggested that in the *MRP*-AS clones, reduced Bcl-2 protein expression was due to a translational or post-translational mechanism.

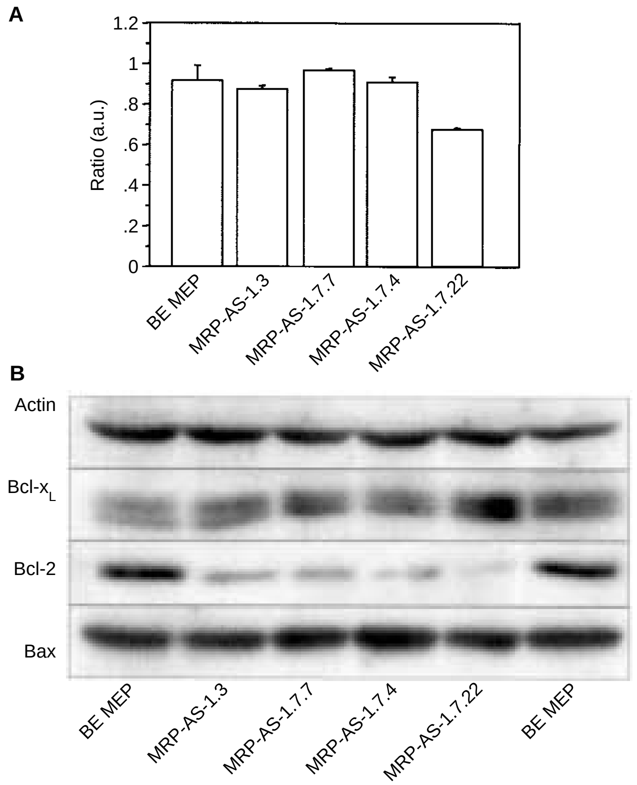

**Figure 7** Expression of Bcl-2 in *MRP*-AS cells. (**A**) Histogram showing the relative expression levels of Bcl-2 mRNA in control and *MRP*-AS cells determined by semi-quantitative RT-PCR as described in the Materials and Methods. Values quantifying mRNA expression are the mean (and standard error of mean) of 3 independent experiments, where the ratio between the densitometric value for *Bcl-2* and the *β-2-microglobulin* PCR products was determined. (**B**) Immunoblots showing protein expression levels of Bcl-2, Bcl-$x_L$, Bax and β-actin in control (BE MEP) and the indicated *MRP*-AS clones

### Ectopic MRP1 overexpression blocks the neuritogenic effects of retinoic acid

The BE cell line was transfected with a vector constitutively overexpressing the full-length human *MRP1* cDNA (BE *MRP*-S). 2 clones (*MRP*-S-540 and *MRP*-S-5512) with an MRP1 expression level 2-fold higher than controls (Figure 1C), were selected for further study. Control (BE CEP) and *MRP*-S cells showed similar morphology and growth characteristics to parental BE cells. To test the function of MRP1 in the *MRP*-S cells, we performed cytotoxicity assays using the MRP1-specific substrate, sodium arsenate (Cole et al, 1994). Compared with controls, *MRP*-S transfectants had a 2.3-fold ($P < 0.05$) higher resistance to the cytotoxic effects of sodium arsenate as measured by $ID_{50}$.

To investigate the effect of increased *MRP1* expression levels on the capacity of NB cells for neuritic differentiation, control and MRP-S cells were treated with aRA. We found that aRA induced a similar level of concentration-dependent growth inhibition in *MRP*-S, BE CEP and BE cells (Figure 8). However, the *MRP*-S clones were markedly resistant to neurite formation (Figure 8). Similar results were observed when the cells were treated with 13-*cis*-retinoic acid or 9-*cis*-retinoic acid (data not shown).

### DISCUSSION

In this study, we have shown that the level of MRP1 protein expression in NB tumour cells has unique effects on cell survival, and on the capacity of NB cells to undergo neuritic differentiation in vitro. Reduced MRP1 expression in *MRP*-AS cells correlated with reduced Bcl-2 protein levels through a post-transcriptional effect on Bcl-2 expression. Taken together, our results suggest the hypothesis that high-level MRP1 expression, may be an acquired characteristic of neuroblasts undergoing malignant transformation in vivo, which contributes to the malignant phenotype by blocking the developmentally regulated neuronal deletion which occurs during embryogenesis of the neural crest.

A link between MRP1 expression level, and protection against cell death caused by substances other than chemotherapeutic cytotoxic drugs, has not been reported. However, recent studies indicate that another membrane-associated efflux pump, Pgp, may protect malignant cells against caspase-dependent apoptosis induced by activation of Fas, serum deprivation, and other death-inducing stimuli (Robinson et al, 1997; Smyth et al, 1998; Johnstone et al, 1999). Growth factors and other morphogens involved in neural crest development are possible MRP1 export substrates. Embryonal neuronal deletion is regulated by multiple neurotrophins in an age- and cell type-specific manner (Davies, 1997). Thus far, basic fibroblast growth factor, acting as a survival factor for Kaposi's and osteogenic sarcoma cells, is the only protein defined as an MRP1 export substrate (Aggarwal and Gupta, 1998; Gupta et al, 1998).

Our studies are the first to link MRP1 expression level with a process of differentiation and cell death. Our previous findings linking MRP1 expression levels in NB primary tumour tissue and patient prognosis, combined with the data presented here, indicate that MRP1 may play a role in the resistance of some NB tumour cells to spontaneous regression. Further in vivo studies are required to substantiate this hypothesis and to define the mechanism of action of MRP1 in NB tumour cells.

© 2001 Cancer Research Campaign

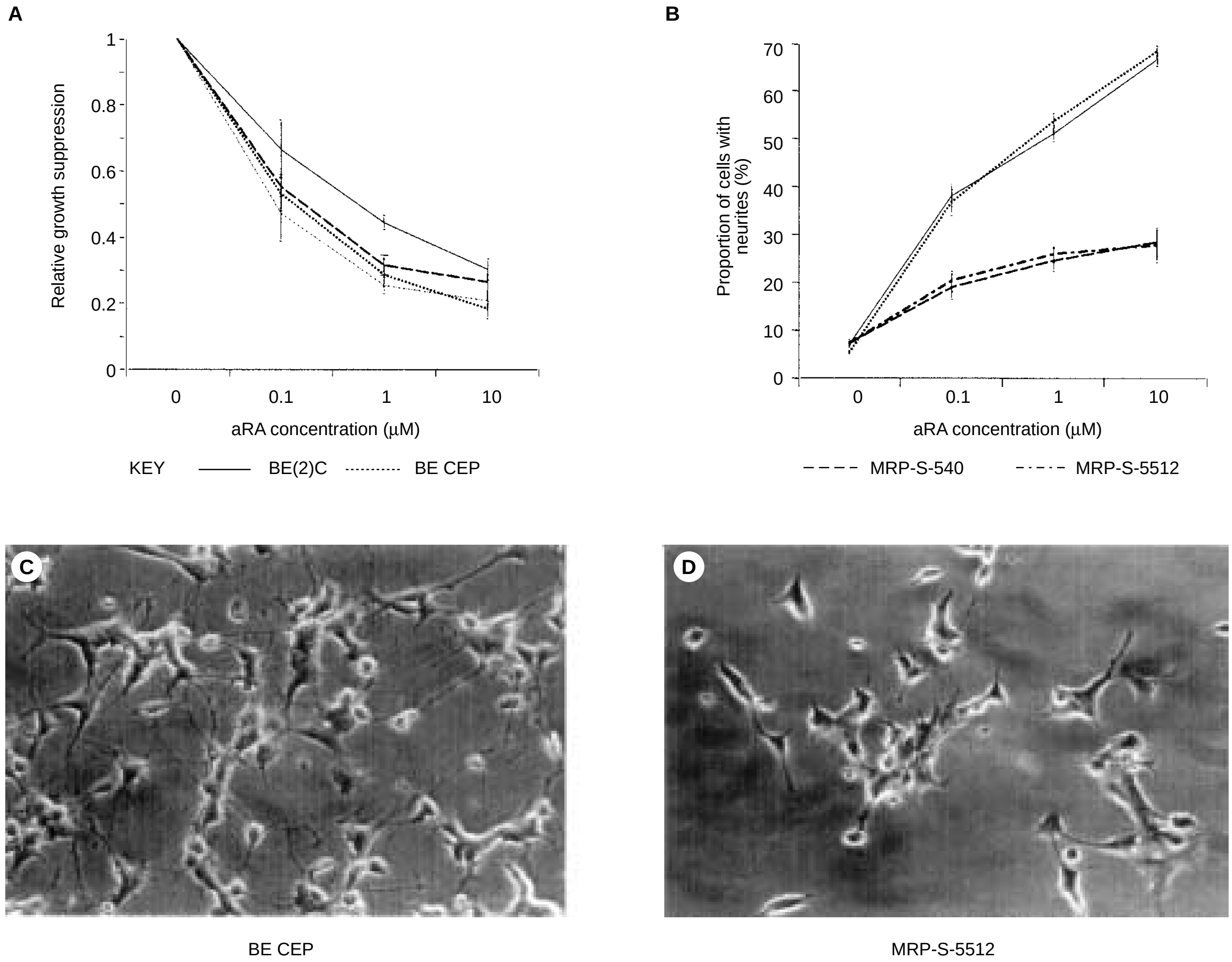

**Figure 8** Growth rate and morphologic response of *MRP*-S transfectants following continuous culture in aRA. (**A**) Control (BE and CEP) and BE *MRP*-S cells (MRP540, MRP5512) were continuously treated with different concentrations of aRA for 7 days, then counted and morphology assessed. Relative growth suppression was calculated by dividing the number of cells present in the treated flask by the cell number present in an untreated flask. (**B**) The same cell lines were assessed for the proportion of cells with neurites after 7 days of continuous culture in different concentrations of aRA. Values represent mean of 2 experiments performed in triplicate. Bars, standard error of mean. *c* and *d*, representative photomicrographs of CEP (**C**) and MRP5512 (**D**) cells, grown in 10 μM aRA for 7 days (100 × magnification)

### ACKNOWLEDGEMENTS

This work was supported by the National Health and Medical Research Council of Australia, the New South Wales State Cancer Council, the Sydney Children's Hospital Foundation and the Children's Cancer Institute Australia. AEP was supported by an Australian Postgraduate Award. Children's Cancer Institute Australia for Medical Research is affiliated with University of New South Wales and Sydney Children's Hospital.

### REFERENCES

Aggarwal S and Gupta S (1998) A possible role of multidrug resistance-associated protein (MRP) in the secretion of basic fibroblast growth factor by osteosarcoma cell line (MG-63). *Int J Oncol* **13**: 1331–1334

Bader P, Schilling F, Schlaud M, Girgert R, Handgertinger R, Klingebiel T, Treuner J, Liu C, Niethammer D and Beck JF (1999) Expression of multidrug resistance associated genes in neuroblastomas. *Oncology Rep* **6**: 1143–1146

Beckwith JB and Perrin EV (1963) In situ neuroblastoma. *Am J Path* **43**: 1089–1104

Bordow SB, Haber M, Madafiglio J, Cheung B, Marshall GM and Norris MD (1994) Expression of the multidrug resistance-associated protein (MRP) gene correlates with amplification and overexpression of the N-myc oncogene in childhood neuroblastoma. *Cancer Res* **54**: 5036–5040

Chomczynski P and Sacchi N (1987) Single-step method of RNA isolation by acid guanidinium thiocyanate-phenol-chloroform extraction. *Anal Biochem* **162**: 156–159

Cole SP, Bhardwaj G, Gerlach JH, Mackie JE, Grant CE, Almquist KC, Stewart AJ, Kurz EU, Duncan AM and Deeley RG (1992) Overexpression of a transporter gene in a multidrug-resistant human lung cancer cell line. *Science* **258**: 1650–1654

Cole SPC, Sparks KE, Fraser K, Loe DW, Grant CE, Wilson GM and Deeley RG (1994) Pharmacological characterisation of multidrug resistant MRP-transfected human tumor cells. *Cancer Res* **54**: 5902–5910

Davies AM (1997) Neurotrophin switching: where does it stand? *Curr Opin Neurobiol* **7**: 110–118

Deeley RG and Cole SPC (1997) Function, evolution and structure of multidrug resistance protein (MRP). *Semin Cancer Biol* **8**: 193–204

Evans AE, Gerson J and Schnaufer L (1976) Spontaneous regression of neuroblastoma. *Natl Cancer Inst Mongr* **44**: 49–54

Grant CE, Valdimarsson G, Hipfner DR, Almquist KC, Cole SPC and Deeley RG (1994) Overexpression of multidrug resistance-associated protein (MRP) increases to natural product drugs. *Cancer Res* **54**: 357–361

Gupta S, Aggarwal S and Nakamura S (1998) A possible role of multidrug resistance-associated protein (MRP) in basic fibroblast growth factor secretion by AIDS-associated Kaposi's sarcoma cells: a survival molecule? *J Clin Immunol* **18**: 256–263

 *© 2001 Cancer Research Campaign*

Haber M, Reed C, Kavallaris M, Norris MD and Stewart BS (1989) Resistance to drugs associated with the multidrug resistance phenotype following selection with high concentration methotrexate. *J Natl Cancer Inst* **81**: 1250–1254

Haber M, Madafiglio J, Bordow SB, Gilbert J, Cheung B, Marshall GM and Norris MD (1994) Expression of retinoic acid-responsive genes in primary neuroblastomas. *Prog Clin Biol Res* **385**: 245–251

Hoehner JC, Hedborg F, Wiklund HJ, Olsen L and Pahlman S (1995) Cellular death in neuroblastoma: in situ correlation of apoptosis and bcl-2 expression. *Int J Cancer* **62**: 19–24

Jahn R, Schiebler W, Ouimet C and Greengard P (1985) A 38,000-dalton membrane protein (p38) present in synaptic vesicles. *Proc Natl Acad Sci USA* **82**: 4137–4141

Johnstone RW, Cretney E and Smyth MJ (1999) P-glycoprotein protects leukemia cells against caspase-dependent, but not caspase-independent, cell death. *Blood* **93**: 1075–1085

Kroemer G (1997) The proto-oncogene Bcl-2 and its role in regulating apoptosis. *Nature Med* **3**: 614–628

Lock RB and Stribinskiene L (1996) Dual modes of death induced by etoposide in human epithelial tumor cells allow Bcl-2 to inhibit apoptosis without affecting clonogenic survival. *Cancer Res* **56**: 4006–4012

Lorico A, Rappa G, Finch RA, Yang D, Flavell RA and Sartorelli AC (1997) Disruption of the murine MRP gene leads to increased sensitivity to etoposide (VP-16) and increased levels of glutathione. *Cancer Res* **57**: 5238–5242

Marshall GM, Cheung B, Stacey KP, Camacho ML, Simpson AM, Kwan E, Smith S, Haber M and Norris MD (1995) Increased retinoic acid receptor gamma expression suppresses the malignant phenotype and alters the differentiation potential of human neuroblastoma cells. *Oncogene* **11**: 485–491

Matsunaga T, Shirasawa H, Hishiki T, Enomoto H, Kouchi K, Ohtsuka Y, Iwai J, Yoshida H, Tanabe M, Kobayashi S, Asano T, Etoh T, Nishi Y and Ohnuma N (1998) Expression of MRP and cMOAT in childhood neuroblastomas and malignant liver tumors and its relevance to clinical behaviour. *Jpn J Cancer Res* **89**: 1276–1283

Norris MD, Bordow SB, Marshall GM, Haber PS, Cohn SL and Haber M (1996) Expression of the gene for multidrug-resistance-associated protein and outcome in patients with neuroblastoma. *N Engl J Med* **334**: 231–238

Robinson LJ, Roberts WK, Ling TT, Lamming D, Sternberg SS and Roepe PD (1997) Human MDR1 protein overexpression delays apoptotic cascade in Chinese hamster ovary fibroblasts. *Biochemistry* **36**: 11169–11178

Shimada H, Chatten J, Newton WA, Sachs N, Hamoudi AB, Chiba T, Marsden HB and Misugi K (1984) Histopathologic prognostic factors in neuroblastic tumors: definition of subtypes of ganglioneuroblastoma and an age-linked classification of neuroblastomas. *J Natl Cancer Inst* **73**: 405–416

Smyth MJ, Krasovskis E, Sutton VR and Johnstone RW (1998) The drug efflux protein, P-glycoprotein, additionally protects drug-resistant tumor cells from multiple forms of caspase-dependent apoptosis. *Proc Natl Acad Sci USA* **95**: 7024–7029

Wijnholds J, Evers R, van Leusden MR, Mol CA, Zaman GJ, Mayer U, Beijnen JH, van der Valk M, Krimpenfort P and Borst P (1997) Increased sensitivity to anticancer drugs and decreased inflammatory response in mice lacking the multidrug resistance-associated protein. *Nature Med* **3**: 1275–1279

Woods WG, Tuchman M, Robison LL, Bernstein M, Leclerc JM, Brisson LC, Brossard J, Hill G, Shuster J, Leupker R, Byrne T, Weitzman S, Bunin G and Lemieux B (1996) A population-based study of the usefulness of screening for neuroblastoma. *Lancet* **348**: 1682–1687

*© 2001 Cancer Research Campaign*

## References

[PDF_00689] Aggarwal S., Gupta S. (1998). A possible role for multidrug resistance-associated protein in the secretion of basic fibroblast growth factor by osteogenic sarcoma cell line (MG-63).. Int J Oncol.

[PDF_00695] BECKWITH J. B., PERRIN E. V. (1963). IN SITU NEUROBLASTOMAS: A CONTRIBUTION TO THE NATURAL HISTORY OF NEURAL CREST TUMORS.. Am J Pathol.

[PDF_00692] Bader P., Schilling F., Schlaud M., Girgert R., Handgretinger R., Klingebiel T., Treuner J., Liu C., Niethammer D., Beck J. F. (1999). Expression analysis of multidrug resistance associated genes in neuroblastomas.. Oncol Rep.

[PDF_00696] Bordow S. B., Haber M., Madafiglio J., Cheung B., Marshall G. M., Norris M. D. (1994). Expression of the multidrug resistance-associated protein (MRP) gene correlates with amplification and overexpression of the N-myc oncogene in childhood neuroblastoma.. Cancer Res.

[PDF_00700] Chomczynski P., Sacchi N. (1987). Single-step method of RNA isolation by acid guanidinium thiocyanate-phenol-chloroform extraction.. Anal Biochem.

[PDF_00703] Cole S. P., Bhardwaj G., Gerlach J. H., Mackie J. E., Grant C. E., Almquist K. C., Stewart A. J., Kurz E. U., Duncan A. M., Deeley R. G. (1992). Overexpression of a transporter gene in a multidrug-resistant human lung cancer cell line.. Science.

[PDF_00706] Cole S. P., Sparks K. E., Fraser K., Loe D. W., Grant C. E., Wilson G. M., Deeley R. G. (1994). Pharmacological characterization of multidrug resistant MRP-transfected human tumor cells.. Cancer Res.

[PDF_00709] Davies A. M. (1997). Neurotrophin switching: where does it stand?. Curr Opin Neurobiol.

[PDF_00711] Deeley R. G., Cole S. P. (1997). Function, evolution and structure of multidrug resistance protein (MRP).. Semin Cancer Biol.

[PDF_00713] Evans A. E., Gerson J., Schnaufer L. (1976). Spontaneous regression of neuroblastoma.. Natl Cancer Inst Monogr.

[PDF_00715] Grant C. E., Valdimarsson G., Hipfner D. R., Almquist K. C., Cole S. P., Deeley R. G. (1994). Overexpression of multidrug resistance-associated protein (MRP) increases resistance to natural product drugs.. Cancer Res.

[PDF_00718] Gupta S., Aggarwal S., Nakamura S. (1998). A possible role of multidrug resistance-associated protein (MRP) in basic fibroblast growth factor secretion by AIDS-associated Kaposi's sarcoma cells: a survival molecule?. J Clin Immunol.

[PDF_00760] Haber M., Madafiglio J., Bordow S. B., Gilbert J., Cheung B., Marshall G. M., Norris M. D. (1994). Expression of retinoic acid-responsive genes in primary neuroblastomas.. Prog Clin Biol Res.

[PDF_00757] Haber M., Reed C., Kavallaris M., Norris M. D., Stewart B. W. (1989). Resistance to drugs associated with the multidrug resistance phenotype following selection with high-concentration methotrexate.. J Natl Cancer Inst.

[PDF_00763] Hoehner J. C., Hedborg F., Wiklund H. J., Olsen L., Påhlman S. (1995). Cellular death in neuroblastoma: in situ correlation of apoptosis and bcl-2 expression.. Int J Cancer.

[PDF_00766] Jahn R., Schiebler W., Ouimet C., Greengard P. (1985). A 38,000-dalton membrane protein (p38) present in synaptic vesicles.. Proc Natl Acad Sci U S A.

[PDF_00769] Johnstone R. W., Cretney E., Smyth M. J. (1999). P-glycoprotein protects leukemia cells against caspase-dependent, but not caspase-independent, cell death.. Blood.

[PDF_00772] Kroemer G. (1997). The proto-oncogene Bcl-2 and its role in regulating apoptosis.. Nat Med.

[PDF_00774] Lock R. B., Stribinskiene L. (1996). Dual modes of death induced by etoposide in human epithelial tumor cells allow Bcl-2 to inhibit apoptosis without affecting clonogenic survival.. Cancer Res.

[PDF_00777] Lorico A., Rappa G., Finch R. A., Yang D., Flavell R. A., Sartorelli A. C. (1997). Disruption of the murine MRP (multidrug resistance protein) gene leads to increased sensitivity to etoposide (VP-16) and increased levels of glutathione.. Cancer Res.

[PDF_00780] Marshall G. M., Cheung B., Stacey K. P., Camacho M. L., Simpson A. M., Kwan E., Smith S., Haber M., Norris M. D. (1995). Increased retinoic acid receptor gamma expression suppresses the malignant phenotype and alters the differentiation potential of human neuroblastoma cells.. Oncogene.

[PDF_00784] Matsunaga T., Shirasawa H., Hishiki T., Enomoto H., Kouchi K., Ohtsuka Y., Iwai J., Yoshida H., Tanabe M., Kobayashi S. (1998). Expression of MRP and cMOAT in childhood neuroblastomas and malignant liver tumors and its relevance to clinical behavior.. Jpn J Cancer Res.

[PDF_00789] Norris M. D., Bordow S. B., Marshall G. M., Haber P. S., Cohn S. L., Haber M. (1996). Expression of the gene for multidrug-resistance-associated protein and outcome in patients with neuroblastoma.. N Engl J Med.

[PDF_00792] Robinson L. J., Roberts W. K., Ling T. T., Lamming D., Sternberg S. S., Roepe P. D. (1997). Human MDR 1 protein overexpression delays the apoptotic cascade in Chinese hamster ovary fibroblasts.. Biochemistry.

[PDF_00795] Shimada H., Chatten J., Newton W. A., Sachs N., Hamoudi A. B., Chiba T., Marsden H. B., Misugi K. (1984). Histopathologic prognostic factors in neuroblastic tumors: definition of subtypes of ganglioneuroblastoma and an age-linked classification of neuroblastomas.. J Natl Cancer Inst.

[PDF_00799] Smyth M. J., Krasovskis E., Sutton V. R., Johnstone R. W. (1998). The drug efflux protein, P-glycoprotein, additionally protects drug-resistant tumor cells from multiple forms of caspase-dependent apoptosis.. Proc Natl Acad Sci U S A.

[PDF_00803] Wijnholds J., Evers R., van Leusden M. R., Mol C. A., Zaman G. J., Mayer U., Beijnen J. H., van der Valk M., Krimpenfort P., Borst P. (1997). Increased sensitivity to anticancer drugs and decreased inflammatory response in mice lacking the multidrug resistance-associated protein.. Nat Med.

[PDF_00807] Woods W. G., Tuchman M., Robison L. L., Bernstein M., Leclerc J. M., Brisson L. C., Brossard J., Hill G., Shuster J., Luepker R. (1996). A population-based study of the usefulness of screening for neuroblastoma.. Lancet.

